# Correlation Between Radiological Changes of the Temporomandibular Joint and Upper Cervical Vertebrae in Degenerative Joint Disease: A Cone-Beam Computed Tomography-Based Analytical Study

**DOI:** 10.7759/cureus.67518

**Published:** 2024-08-22

**Authors:** Ajay G Nayak, Sunanda Bhatnagar, Atrey J Pai Khot

**Affiliations:** 1 Department of Maxillofacial Imaging, INsight CBCT (Cone-Beam Computed Tomography) Imaging Center, Mumbai, IND; 2 Department of Oral Medicine and Radiology, Terna Public Charitable Trust's Terna Dental College, Navi Mumbai, IND; 3 Department of Public Health Dentistry, Goa Dental College and Hospital, Bambolim, IND

**Keywords:** temporomandibular joint (tmj), upper cervical spine, severity of disease, diagnostic ct imaging, degenerative joint disease, cone-beam computed tomography (cbct)

## Abstract

Objectives

This study was conducted to assess the radiological changes of the temporomandibular joint (TMJ) and cervical vertebrae individually and their correlation in degenerative joint disease (DJD) using a cone-beam computed tomography (CBCT)-based approach.

Methodology

The study employed a cross-sectional, analytical retrospective design, analyzing one-year data. CBCT scans of 60 patients (120 TMJs) were assessed for degenerative changes using standardized imaging parameters. Eligibility criteria included full field-of-view CBCT scans, excluding those with craniofacial anomalies or prior orthodontic treatment. Radiological assessments of TMJs and cervical vertebrae were conducted by experienced radiologists using the Anjos Pontual method and novel grading system (TMJ Spine Degenerative Severity Index).

Results

The study included 60 CBCT scans (120 joints), with 31.7% males and 68.3% females. Participants were predominantly aged 31-60 years (58.3%). DJD findings for the right TMJ showed grade 1 changes in 55.0% and grade 2 in 31.7%, while the left TMJ had 46.7% grade 1 and 35.0% grade 2 changes. A strong positive correlation (0.704) was found between bilateral TMJ and cervical vertebrae changes. Age correlated significantly with TMJ alterations but not with cervical vertebrae changes.

Conclusion

This study demonstrated that there exists a positive association between the radiological changes of TMJ and cervical vertebrae in DJD with age, which can be detected in mild stage of severity on CBCT and can be of use in clinical correlation and application of optimal interventions ensuring better prognosis.

## Introduction

Degenerative joint disease (DJD), also known as osteoarthritis or degenerative arthritis, is a multi-factorial condition that affects the synovial joints. The most commonly affected joints are the load-bearing bones such as the hip and knee, and in the head and neck region, certainly, the cervical spine and the temporomandibular joint (TMJ) [1]. The TMJ is a diarthrodial hinge joint featuring a fibrocartilaginous disc between two articulating surfaces. Recognized as one of the most complex joints in the body, it facilitates rotations and translations essential for activities such as chewing, speaking, and breathing, while enduring significant and repeated forces [2]. The TMJ disc is primarily made of collagen type 1, with its articular surface containing growth plates and the mandibular condyle housing a unique fibrous zone [3]. Degenerative bone changes in the TMJ are significantly more frequent in the condyle than in the articular eminence, and are characterized by the presence of flattening, flat bony contour of the condyle deviating from the convex form; erosion, decreased cortical bone density extending into the bone marrow; and pseudocyst, well-circumscribed osteolytic adjacent subcortical bone area without cortical destruction. These findings are considered to be radiographic signs of osteoarthritis [3,4].

DJD is characterized by degeneration of the articulating cartilage that covers the osseous components of the joint leading to eventual deterioration of the subchondral bone [5]. DJD is considered a sequel of an overuse injury that causes abnormal load distribution and abnormal stresses that are not sufficient to cause a fracture [6,7]. Over time, however, overuse injury gradually causes deterioration of the disc and bone. Therefore, when DJD affects the cervical spine, it usually affects vertebrae C5-C7, where the stresses are greatest, and then decreases in frequency toward vertebrae C2-C3 [8,9]. Imaging features of cervical vertebrae DJD include erosion, osteophyte formation, joint space narrowing, subchondral cysts, joint mice, flattening of the articulating surfaces, and subchondral sclerosis [10]. TMJ osteoarthritis affects the cartilage, subchondral bone, synovial membrane, and other hard and soft tissues causing changes such as TMJ remodeling, articular cartilage abrasion, and deterioration [11]. The cervical spine discs gradually break down, become dehydrated, and stiffen with age. The cervical spine components affected by osteoarthritis are articular cartilage, synovium, uncovertebral joints, facet joints, intervertebral discs and ligaments, and cervical plexus [12]. The disease process is characterized by deterioration and abrasion of articular cartilage and soft tissue surfaces, the occurrence of thickening and remodeling of the underlying bone, and the formation of marginal spurs and sub-articular "cysts" [13]. Clinical examination often fails to detect degenerative bone changes accurately; therefore, radiographic examination is performed to aid in their diagnosis and treatment [14]. The bony components are best detected by computed tomography (CT). Cone-beam computed tomography (CBCT) uses less radiation than multi-slice CT and provides a 3D image of the mineralized maxillofacial tissue with minimum distortion. CBCT has been efficient in the diagnosis of several bone changes in the TMJ [15,16].

The literature revealed the existence of CT/MRI-based severity grading systems for lumbar DJD used for pre-non-fusion spinal surgery [17]. These scoring or classification systems and qualitative enumeration of radiographic features of osteoarthritis cited in the literature could not substantiate to correlate and grade disease severity, disabling adequate treatment evaluation and execution. There was found to be a need for a more structured, standardized semblance for easy classification that would put diagnostic findings into a standardized format, which could simplify radiographic documentation and collaboration with multidisciplinary specialists. This study was conducted to assess TMJ and upper cervical vertebrae in DJD using a CBCT-based retrospective approach. The study aims to assess the correlation between radiological changes in TMJ and upper cervical vertebrae in DJD as well as to evaluate these individually to find the possible association between various descriptive demographic variables such as age, gender, and severity with increasing age. The study also aimed to determine the correlation between changes in the upper cervical vertebrae and unilateral versus bilateral TMJ changes. While doing so, the study evaluated the potential of the CBCT scans in assessing for the same. This clinically applicable classification system would define therapeutic implications more precisely and evaluate objectively any improvements or setbacks over the course of the disease.

## Materials and methods

Study design and ethical consideration

The study followed the design of a cross-sectional, analytical retrospective study and assessed one-year retrospective data from December 1, 2022 to December 1, 2023. The study was conducted in accordance with the Declaration of Helsinki after approval from the institutional ethics committee (TDC/EC/22/2023).

Sample size and source of data

The sample size was estimated using OpenEpi version 3.04 software. Depending on the data formulation, a sample size of 60 was derived. Hence, CBCT scans were obtained for 60 patients who presented to the department of radiology at a tertiary care private diagnostic facility in a metropolitan city in India. These patients were scanned for both right and left TMJs, equaling 120 joints.

Eligibility criteria

Para-sagittal and coronal sections were selected for TMJ assessment and axial and coronal sections were selected for cervical vertebrae assessment. Full field of view CBCT scans of patients aged 18-70 years, CBCT scans in which the occipital condyles and at least C1-C2 vertebrae are entirely captured in the image volume, and CBCT scans with maximum intercuspation were included in the study whereas craniofacial anomalies, orthodontically treated patients, the scans of patients with pathologies like tumor lesions and fractures in the dentomaxillofacial region that could affect the size of the anatomical structures in the TMJ and aplasia or TMJ malformation were excluded from the study.

Assessment and examination procedure

The examination was done using the iCAT 17-19 CBCT Scanner (Imaging Sciences International, Hatfield, PA). Exposure parameters like 120 kVp, field-of-view (FOV) of 17 x 23 cm, size, centering, and voxel size setting (0.25 mm) were standardized according to the purpose of the CBCT scan. The images were assessed twice by two experienced radiologists, with more than 14 years of experience, at an interval of at least 15 days between the assessments of the same scan. Demographic details (age and gender) were recorded. However, names and other identifying details were kept confidential.

**Figure 1:**
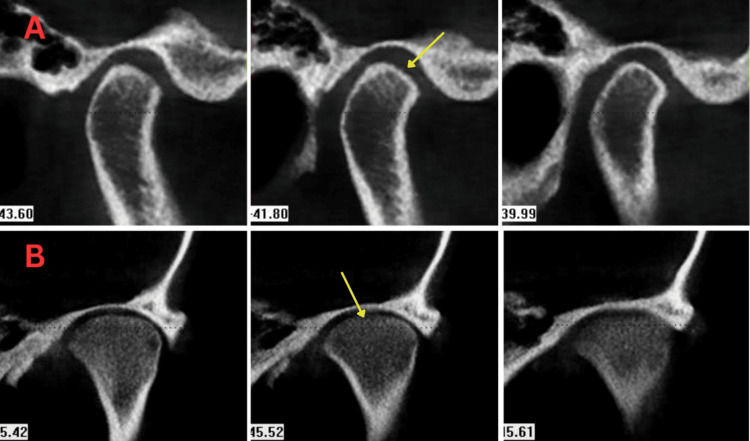
CBCT imaging showing TMJ grade 1. A: Mild flattening of contours. B: Mild thinning of cortical outline. CBCT: cone-beam computed tomography; TMJ: temporomandibular joint.

**Figure 2:**
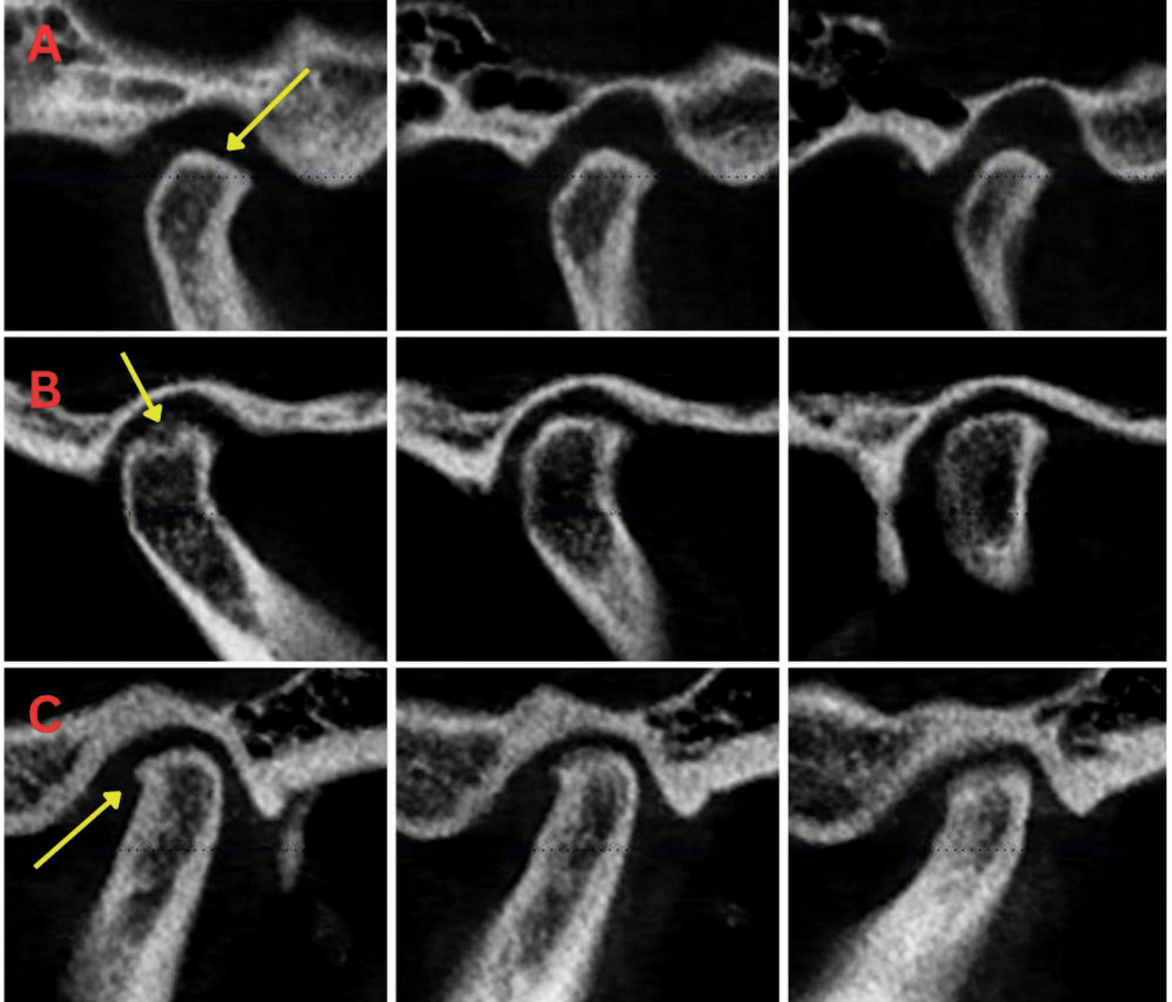
CBCT imaging showing TMJ grade 2. A: Moderate flattening of contours. B: Less than three erosions. C: Small osteophyte. CBCT: cone-beam computed tomography; TMJ: temporomandibular joint.

**Figure 3:**
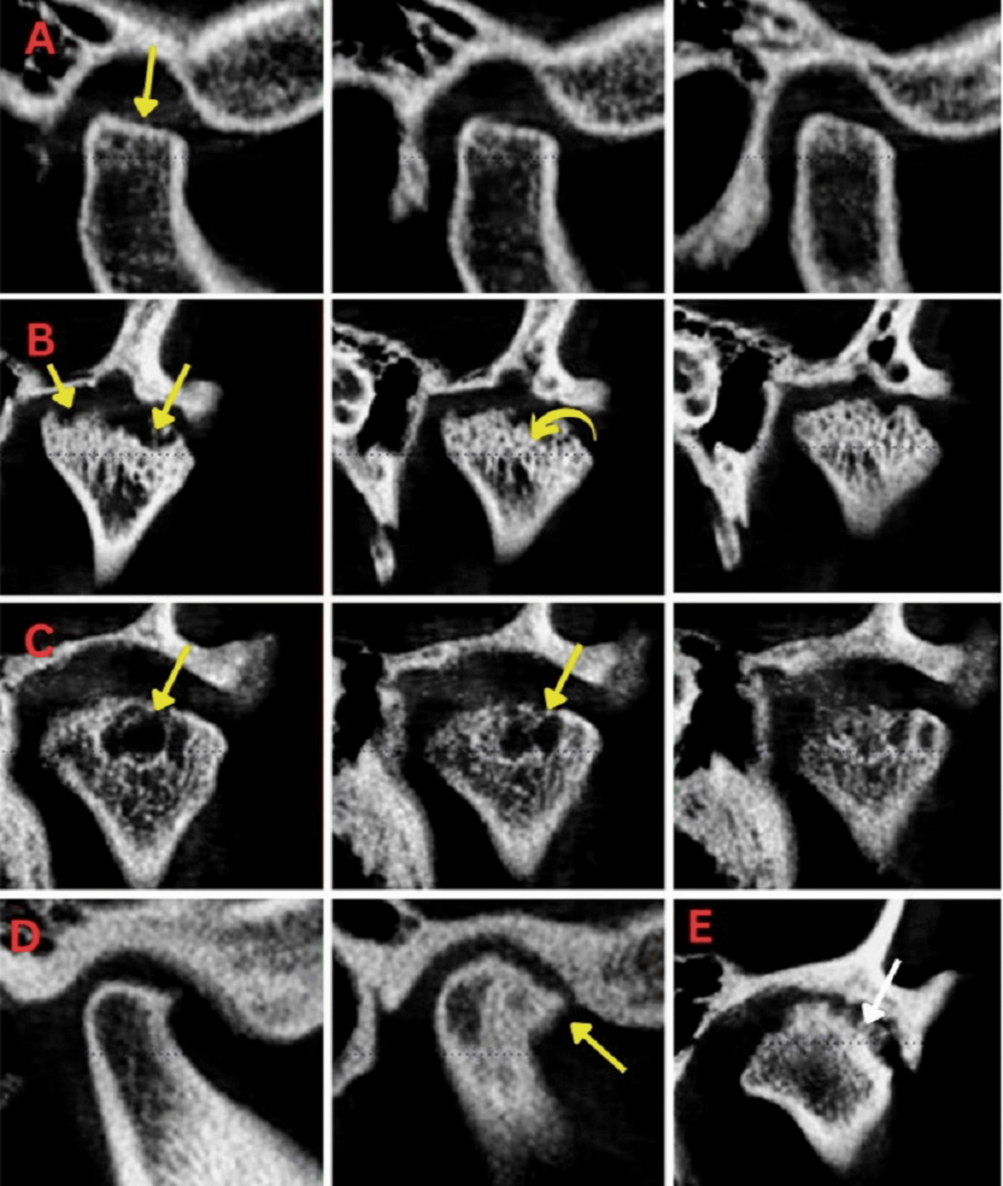
CBCT imaging showing TMJ Grade 3 A: Severe flattening of contours. B: Severe or multiple erosions (more than three). C: Large pseudocyst formation. D: Large osteophyte. E: Severe sclerosis of cortical outline. CBCT: cone-beam computed tomography; TMJ: temporomandibular joint.

**Figure 4:**
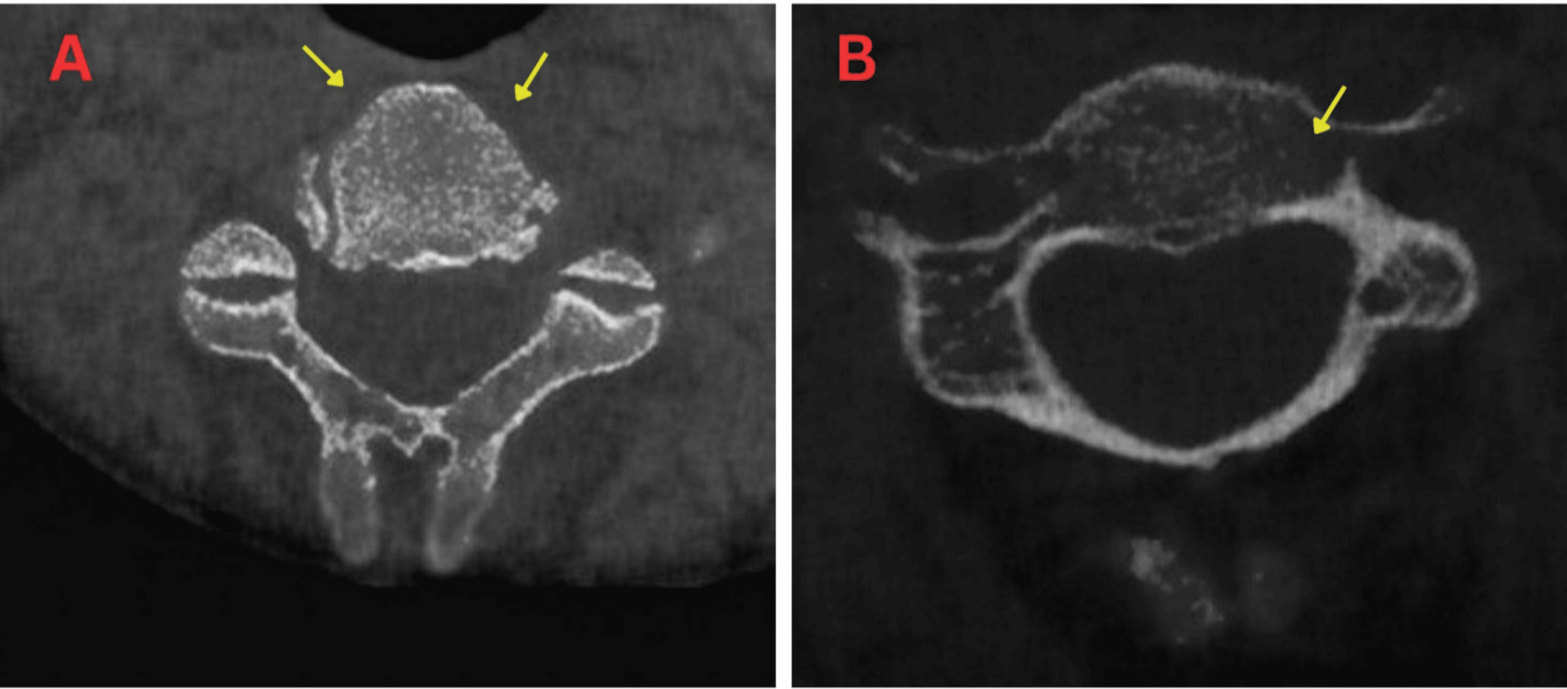
CBCT imaging showing upper cervical vertebrae grade 1. A: Mild thinning of cortical outline. B: Mild rarefaction of the trabecular pattern. CBCT: cone-beam computed tomography.

**Figure 5:**
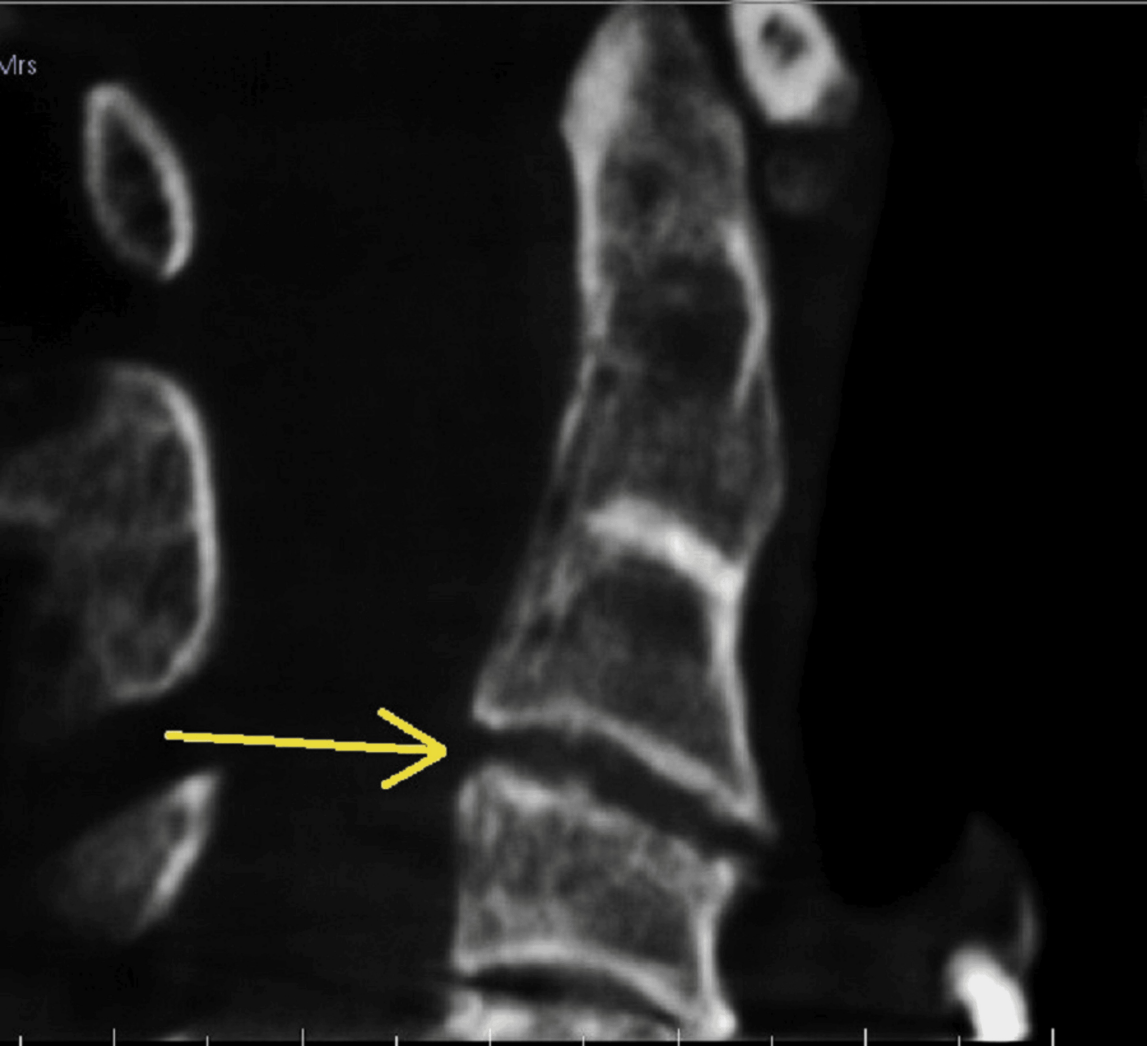
CBCT imaging showing upper cervical vertebrae grade 2. Less than three erosions and small osteophytes. CBCT: cone-beam computed tomography.

**Figure 6:**
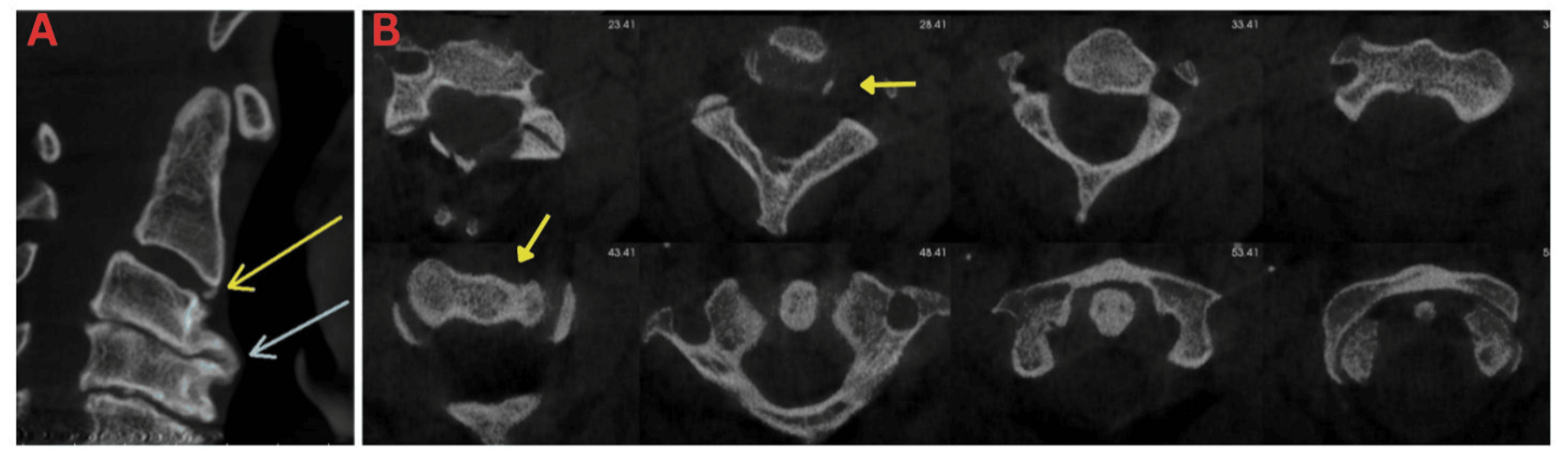
CBCT imaging showing upper cervical vertebrae grade 3. A: Large osteophyte, severe sclerosis of cortical outline, and severe narrowing of intervertebral joint spaces. B: Presence of C1-C2 rotation. CBCT: cone-beam computed tomography.

The scans were assessed for the presence or absence of radiological degenerative changes in both the TMJs and their severity by using the Anjos Pontual method [3]. The novel classification system, CBCT-based grading system for severity in DJD of TMJ and upper cervical spine, is depicted in Tables 1, 2 and Figures 1-6.

**Table 1:** TMJ-Spine Degenerative Severity Index (TMJ component). Table credits: Sunanda Bhatnagar and Ajay G. Nayak. TMJ: temporomandibular joint; DJD: degenerative joint disease.

Grade	Features
0	Normal with no osseous DJD changes (all contours appear curved with no flattening and intact cortical outline with no thinning, sclerosis, or erosions)
1	Mild flattening of contours and mild thinning of cortical outline
2	Moderate flattening of contours, less than three erosions, pseudocyst formation, small osteophyte, mild to moderate sclerosis of cortical outline
3	Severe flattening of contours, severe or multiple erosions (more than three), large pseudocyst formations, large osteophyte, severe sclerosis of cortical outline

**Table 2:** TMJ-Spine Degenerative Severity Index (upper cervical vertebrae (C1-C3) grading component). Table credits: Sunanda Bhatnagar and Ajay G. Nayak. TMJ: temporomandibular joint; DJD: degenerative joint disease.

Grade	Features
0	Normal with no osseous DJD changes (continuous cortical outline with no thinning, sclerosis, or erosions, normal trabecular pattern, and no narrowing of the intervertebral joint spaces)
1	Mild thinning of cortical outline, mild rarefaction of the trabecular pattern
2	Moderate rarefaction of trabecular pattern, less than three erosions, small osteophyte, mild to moderate sclerosis of cortical outline, mild to moderate narrowing of intervertebral joint spaces
3	Severe thinning of cortical outline and severe rarefaction of trabecular pattern, more than three erosions, large osteophyte, severe sclerosis of cortical outline, severe narrowing of intervertebral joint spaces, presence of C1-C2 rotation

Grading system

The scans/joints were graded according to the severity of DJD using our own grading system, and a radiological diagnosis was established. Acknowledging the challenge of distinguishing between normal morphological variations and minor pathological changes, it was decided that in cases of disagreement between the two radiologists regarding the severity grade, the lower grade would be selected after discussion amongst both authors to avoid overestimating the disease. The kappa statistics revealed a substantial level of agreement between the radiologists (0.81, 0.83).

Data collection and statistical analysis

The data were coded into Microsoft Office Excel (Microsoft Corporation, Redmond, WA) and entered into SPSS version 17 software (SPSS Inc., Chicago, IL). The normality of the data was assessed before analysis using Shapiro-Wilk's test. The inter and intra-observer reliability of the data was also calculated using kappa statistics. Descriptive analysis through frequency distribution was calculated and a chi-square test was applied where p‐values of less than 0.05 were considered significant.

## Results

**Figure 7:**
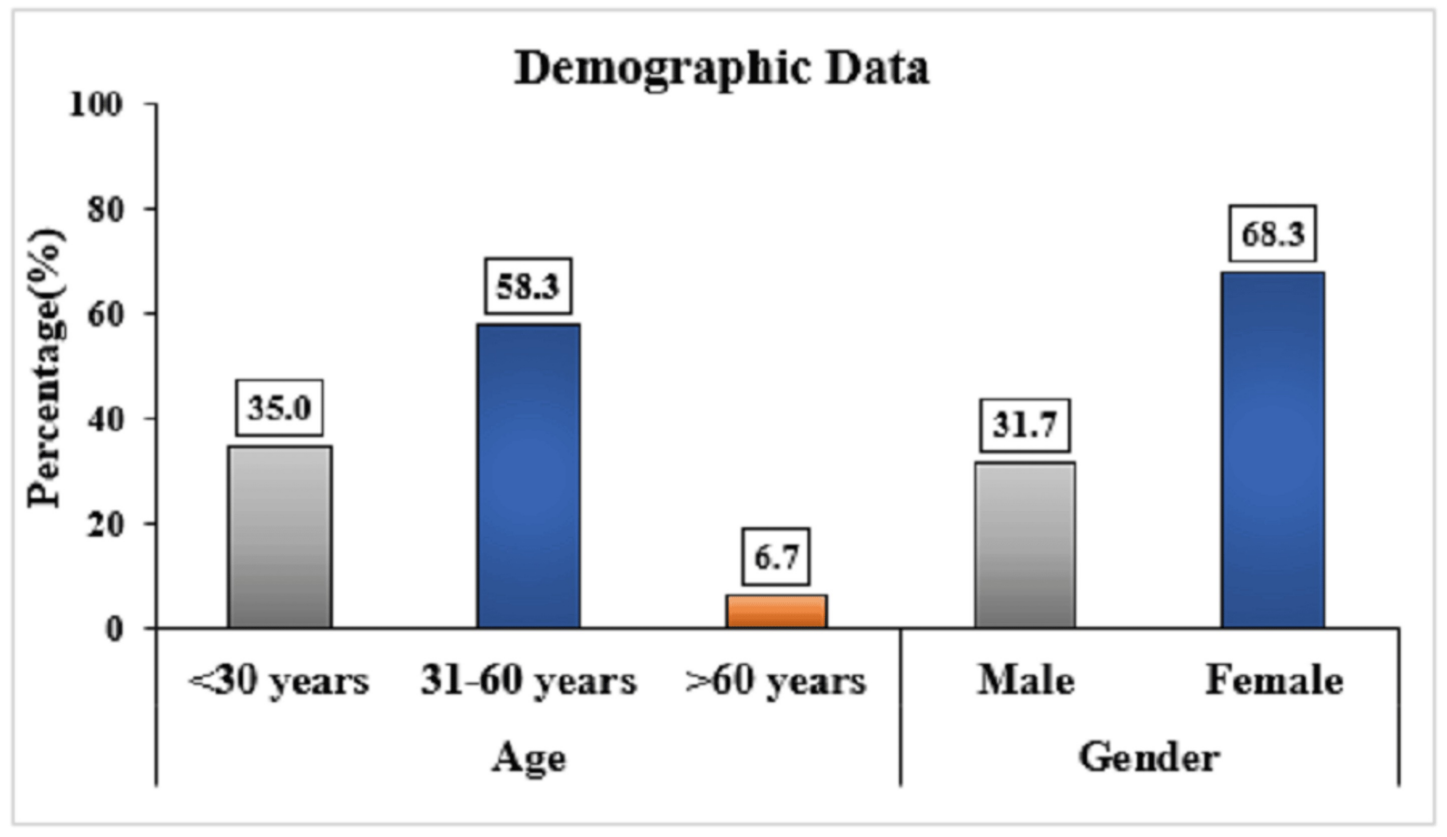
Demographic characteristics of the participants.

A total of 60 scans were included in this study, comprising 19 males (31.7%) and 41 females (68.3%). There were 21 participants (35.0%) under 30 years old, 35 participants (58.3%) were aged between 31 and 60 years, and four participants (6.7%) were over 60 years old. This demographic profile, as depicted in Figure 7, reflects a majority of female participants and a higher representation of individuals aged between 31 and 60 years.

The majority of the participants, which included 33 individuals (55.0%), showed grade 1 findings in the right TMJ. The least common observation was grade 3 findings, which were present in only one participant (1.7%). Similarly, the majority of participants, 28 individuals (46.7%), showed grade 1 findings. The frequency of DJD findings in relation to the right and left TMJs has been depicted in Table 3.

**Table 3:** DJD findings in relation to the right and left TMJs. All values are expressed as frequency with percentages (in parentheses). TMJ: temporomandibular joint; DJD: degenerative joint disease.

Finding	Right TMJ, N (%)	Left TMJ, N (%)
Grade 0: Normal joint width with no DJD changes	7 (11.7%)	8 (13.3%)
Grade 1: Mild joint space narrowing, small osteophytes &/or rarefactions/thinning/flattening, articular process hypertrophy	33 (55.0%)	28 (46.7%)
Grade 2: Narrowing of joint space, sclerosis, moderate osteophytes &/or bone erosions, pseudocysts	19 (31.7%)	21 (35.0)
Grade 3: Severe arthritic changes, large osteophyte, narrowing of joint space, sclerosis, subchondral cysts	1 (1.7%)	3 (5.0%)

Table 4 describes the correlation between radiographic changes in unilateral/bilateral TMJ and cervical vertebrae. The Spearman correlation coefficient between radiographic changes in the unilateral TMJ and cervical vertebrae is 0.407, indicating a weak positive correlation, whereas in bilateral TMJ and cervical vertebrae, it was found to be 0.704, which was statistically significant, indicating a strong positive correlation.

**Table 4:** Correlation between radiographic changes in unilateral/bilateral TMJ and cervical vertebrae. The statistical test used was the Spearman correlation test. * P ≤ 0.05 is considered statistically significant. TMJ: temporomandibular joint.

		Unilateral TMJ changes	Bilateral TMJ changes
Cervical vertebrae	Spearman correlation	0.407	0.704
	P-value	0.038*	0.002*

Among the observations in the cervical vertebrae, as depicted in Table 5, the majority of participants, 39 individuals (65.0%), exhibited grade 0 findings, indicating a normal joint width with no DJD changes.

**Table 5:** Frequency of DJD in relation to cervical vertebrae. All values are expressed as frequency with percentages (in parentheses). DJD: degenerative joint disease.

Finding	Cervical vertebrae, N (%)
Grade 0: Normal joint width with no DJD changes	39 (65.0%)
Grade 1: Mild joint space narrowing, small osteophytes &/or rarefactions/thinning, articular process hypertrophy	8 (13.3%)
Grade 2: Narrowing of joint space, sclerosis, moderate osteophytes &/or bone erosions, C1-C2 rotations	13 (21.7%)

The mean age ± standard deviation (SD) of the participants with rarefaction findings was 39.44 ± 9.91 years. For participants exhibiting subchondral sclerosis, the mean age was 36.00 ± 9.89 years. Additionally, participants with C1-C2 rotations had a mean age of 37.67 ± 8.86 years. The frequency of specific cervical vertebrae findings relative to age, with the findings categorized as rarefaction, subchondral sclerosis, and C1-C2 rotations has been depicted in Table 6.

**Table 6:** Frequency of specific cervical vertebrae findings relative to age. All values are expressed as mean ± SD (standard deviation).

Finding	Age (mean ± SD)
Rarefaction	39.44 ± 9.91
Subchondral sclerosis	36.00 ± 9.89
C1-C2 rotations	37.67 ± 8.86

Table 7 reveals a significant correlation between age and TMJ alterations, particularly in the left TMJ. Participants under 30 years old exhibited a notably higher prevalence of grade 0 changes in the left TMJ compared to other age groups (p < 0.001), indicating a normative joint width without DJD changes. Conversely, individuals over 60 years old demonstrated a significantly elevated occurrence of grade 2 changes in the left TMJ (p < 0.05), characterized by joint space narrowing, sclerosis, and moderate osteophytes, indicative of more advanced DJD. There was no statistically significant difference obtained between age, gender, and upper cervical vertebrae changes.

**Table 7:** Association of radiographic changes of TMJ and cervical vertebrae with demographics. All values are expressed as frequency and percentage. The statistical test used was the chi-square test. * P ≤ 0.05 is considered statistically significant. TMJ: temporomandibular joint.

Variables	Age					Gender			
	<30 years, N (%)	31-60 years, N (%)	>60 years, N (%)	X^2^	P-value	Male, N (%)	Female, N (%)	X^2^	P-value
	Right TMJ								
Grade 0	4 (19.0%)	3 (8.6%)	0 (0.0%)	4.513	0.608	1 (5.3%)	6 (14.6%)	3.178	0.365
Grade 1	9 (42.9%)	22 (62.9%)	2 (50.0%)			11 (57.9%)	22 (53.7%)		
Grade 2	8 (38.1%)	9 (25.7%)	2 (50.0%)			6 (31.6%)	13 (31.7%)		
Grade 3	0 (0.0%)	1 (2.9%)	0 (0.0%)			1 (5.3%)	0 (0.0%)		
	Left TMJ								
Grade 0	5 (23.8%)	3 (8.6%)	0 (0.0%)	25.197	<0.001*	3 (15.8 %)	5 (12.2%)	2.426	0.489
Grade 1	12 (57.1%)	16 (45.7%)	0 (0.0%)			7 (36.8%)	21 (51.2%)		
Grade 2	4 (19.0%)	15 (42.9%)	2 (50.0%)			7 (36.8%)	14 (34.1%)		
Grade 3	0 (0.0%)	1 (2.9%)	2 (50.0%)			2 (10.5%)	1 (2.4%)		
	Cervical vertebrae								
Grade 0	16 (76.2%)	19 (54.3%)	4 (100.0%)	5.832	0.212	13 (68.4%)	26 (63.4%)	0.619	0.734
Grade 1	1 (4.8%)	7 (20.0%)	0 (0.0%)			3 (15.8%)	5 (12.2%)		
Grade 2	4 (19.0%)	9 (25.7%)	0 (0.0%)			3 (15.8%)	10 (24.4%)		
Grade 3	0 (0.0%)	0 (0.0%)	0 (0.0%)			0 (0.0%)	0 (0.0%)		

## Discussion

DJD is a complex condition characterized by the deterioration of joint structures, making it one of the most prevalent pathological issues affecting a variety of joints throughout the body, including the TMJ and cervical vertebrae. It has been observed that there exists an intersection of DJD in TMJ and upper cervical vertebrae that may affect the overall disease severity and treatment execution [5]. Numerous studies in the literature have done morphometric analysis of joint space and have classified joint space narrowing [18]. Gadolinium-enhanced MRI continues to have an unquestioned reputation for providing highly specific and sensitive diagnoses [19,20]. However, it cannot be used for routine screening and early detection due to its invasiveness, limited availability, and high cost as compared to CBCT [21]. Ultrasonography (USG), in particular, is a desirable diagnostic modality being non-invasive and inexpensive. Nevertheless, USG does not provide sufficient sensitivity and is only trustworthy for identifying extremely advanced stages of change [22]. However, the condylar position and the measurements associated with it have been a controversial subject attracting much attention because many variables will influence the condylar position when CBCT is used for the TMJ examination and one should be careful to use the joint space as a diagnostic criterion, at least as the only criterion [23,24]. CBCT as an imaging modality versus other modalities like CT and micro CT has been a point of analysis for researchers who have concluded that CBCT most accurately depicts erosive changes of the bone cortex of the mandibular condyle and high detectability was confirmed [24]. The sensitivity of CBCT in the diagnosis of osseous changes in the TMJ is dependent on factors like the size of the defect, large versus standardized view, and resolution based on voxel size. It was found that regardless of the size of the defect and the viewing protocol, the osseous defects could be detected with more than 80% sensitivity. However, for small voxel size of scanning resolution, sensitivity was improved [25]. The existing studies cited in the literature were based on CT as the imaging modality. The present study chose CBCT for imaging of TMJ and upper cervical vertebrae owing to the clear visualization together with higher resolution and lower radiation exposure that it offers for both the anatomical structures lying in close vicinity to each other [26].

The present study findings reveal a complex relationship between DJD and both the TMJs and upper cervical vertebrae. The analysis showed a higher prevalence of DJD changes in the TMJ among older participants, with a notable trend of increasing severity in left TMJ as age progressed. This aligns with existing literature on the progressive nature of joint degeneration with age, emphasizing the importance of age as a significant factor influencing the severity and progression of DJD [27,28]. In recent times, it has been observed that the prevalence of DJD in the younger population may be attributed to the chronic posture associated with the increasing use of handheld electronic devices [29]. Neck flexing and abnormal posture for an extended period may increase stress on the cervical spine [5,12]. It was found that prolonged usage of handheld electronic devices (more than four hours a day), could negatively affect, both posture and respiratory function, which in turn leads to increased stress to the upper cervical spine. This further explains the variations seen in the present scenario, both in clinical presentation and correlating radiological changes, in TMD and cervical vertebrae as compared to earlier times [30]. However, in this study, no significant associations were found between TMJ and cervical vertebrae alterations with age or gender, despite age being a known risk factor for cervical spine degeneration. This implies that other factors beyond age and gender may play more prominent roles in the development and progression of degenerative changes in the TMJ and cervical spine. It is interesting to note that in a study, it was found that DJD of TMJ occurred more frequently amongst women, in bilateral locations and there was an increase in alterations with age [31]. These results underscore the progressive nature of degenerative processes in the TMJ, with age serving as a pivotal factor influencing disease severity and progression. However, another study concluded that DJD of the cervical spine equally affects both genders with increasing age [14]. In another study evaluating the prevalence of osteoarthritis, it was found that females are more commonly affected as compared to males [32].

A study was conducted to evaluate the association of neck pain with TMJ dysfunction in the adult population, wherein it was found that there is a significant association between neck pain and temporomandibular symptomatology [33]. This necessitated further analysis of the correlation between the two structures, which the present study attempted to evaluate. In the present study, the correlation analysis indicated a minimal linear relationship between radiographic changes in the TMJ and those in the cervical vertebrae. These findings suggest that while there may be a weak or non-existent correlation between the degeneration in the TMJ and cervical spine, other factors such as biomechanical or physiological distinctions between the regions could be influencing this lack of correlation [34]. The present study involves a qualitative assessment of the radiological findings and refrains from any quantitative assessment of joint spacing, etc. This is owing to the fact that the measurements made on any single slice are essentially in a 2D plane. This slice thickness can be varied as per the software operator and cannot be fixed or standardized at a particular level. If a slice is 1 mm in thickness, the measurement made on that slice cannot be considered representative or extrapolated to the rest of the joint which can be more than 1 cm wide. The condylar head as well as the glenoid fossa are curved 3D structures that will not share the distance uniformly at all their points throughout the entire joint space [34,35]. The angle of the slice can also vary and give differing measurements. Although the scan is 3D, giving measurements on a 2D slice does not justify scientific correctness. More importantly, these measurements and slices cannot be replicated, hence, leading to questionable validity in our opinion.

The limitation of the study is that it did not include healthy TMJs, which are ideal controls to circumvent unnecessary CBCT radiation. Therefore, no comparison was drawn between the radiographic changes observed versus normalcy. The study was done on a restrictive sample size without clinical correlation, thus inhibiting effective clinical decision-making, and it cannot be extrapolated or generalized to a larger mixed population. Thereby, laying down the need for a prospective study in the future. The CBCT grading system used in this study for the severity of DJD can serve as a simple and standardized collated system of assessment enabling efficient correlation of radiological changes with clinical findings. This innovative CBCT-based radiological tool can be used for the primary assessment of TMJ and upper cervical vertebrae to further aid in a better understanding of the pathology, diagnosis, and treatment execution. It is a comprehensive grading system as it includes all the documented radiological changes that occur and also explains the severity of DJD in both TMJ and upper cervical vertebrae. The futuristic scope of this study involves the clinical correlation of the radiographic findings of the DJD of the TMJ and cervical vertebrae that corresponds to the treatment modalities required and their execution. Any further study conducted in varied age groups and a larger, mixed population may be of great value addition to the existing knowledge and may help refine clinical diagnosis and treatment planning in these cases.

## Conclusions

This study concludes that there exists a positive association between the radiological changes of TMJ and cervical vertebrae in DJD with age, which can be detected in the mild stage of severity on CBCT and can be of use in clinical correlation and application of optimal interventions ensuring better prognosis. This grading system is user-friendly and easy to comprehend and apply in radiological interpretation. The application of this novel grading system can also be imperative and of paramount importance for efficient pre- and post-management assessment of patients. This would contribute to a more streamlined treatment protocol that can be standardized according to the grade of severity. The classification system used can serve as a stepping stone in providing modifications to existing treatment modalities.
